# Supplementary material for: Microbially induced calcite precipitation using Bacillus velezensis with guar gum
Source: PLoS One. 2020 Aug 12;15(8):e0236745. doi: 10.1371/journal.pone.0236745 (PMC7423064; doi:10.1371/journal.pone.0236745)
Supplement: S1 File — (PDF) [file pone.0236745.s001.pdf]

<u>**Supplementary Material for**</u>

Microbially induced calcite precipitation using *Bacillus velezensis* with guar gum

Rashmi Dikshit[a], Animesh Jain[a], Arjun Dey[b], Aloke Kumar[a*]

[a]Department of Mechanical Engineering, Indian Institute of Science, Bangalore-560012, India

[b]Thermal Systems Group, U. R. Rao Satellite Centre (Formerly known ISRO Satellite Centre), Indian Space Research Organisation, Bangalore-560017, India.

[*]Corresponding author email id: alokekumar@iisc.ac.in

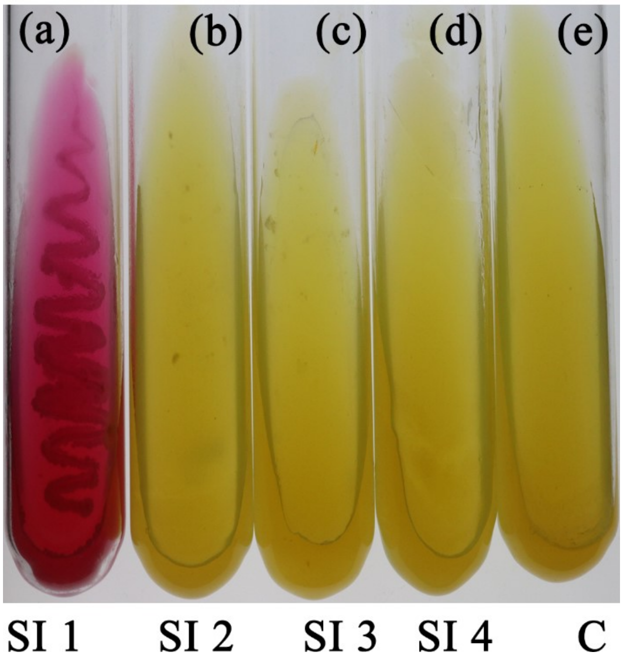

S1 Fig. Agar column showing urea hydrolysis after 24h of Soil Isolated strains (a) SI1, (b) SI2, (c) SI3, (d) SI4, (e) C (without organism). SI1 shows colour change due to increase in medium pH by bacterial activity.SI1; soil isolate 1, SI2; soil isolate 2, SI3; soil isolate 3, SI4; soil isolate 4.

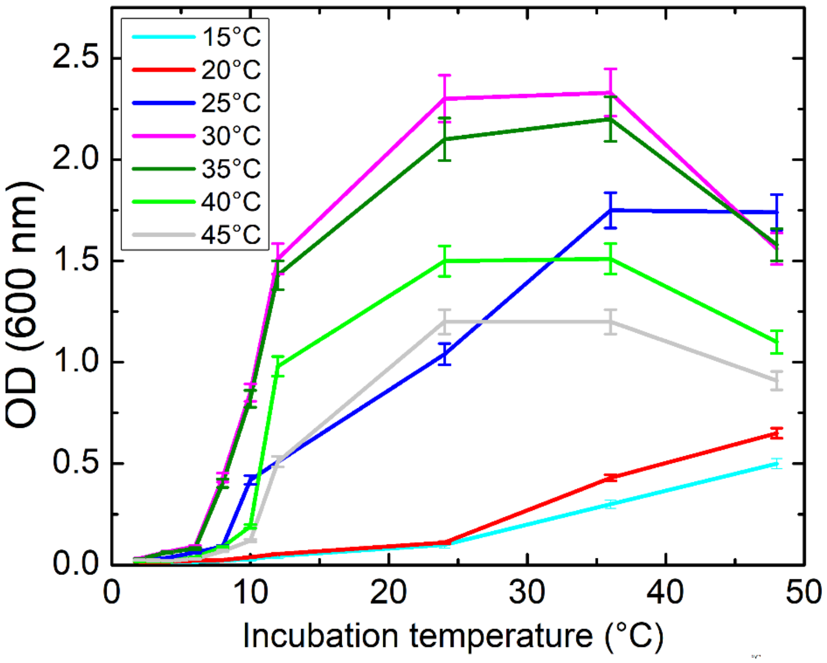

S2 Fig. Temporal evolution of bacterial growth under varied range of temperature (15-45 °C).

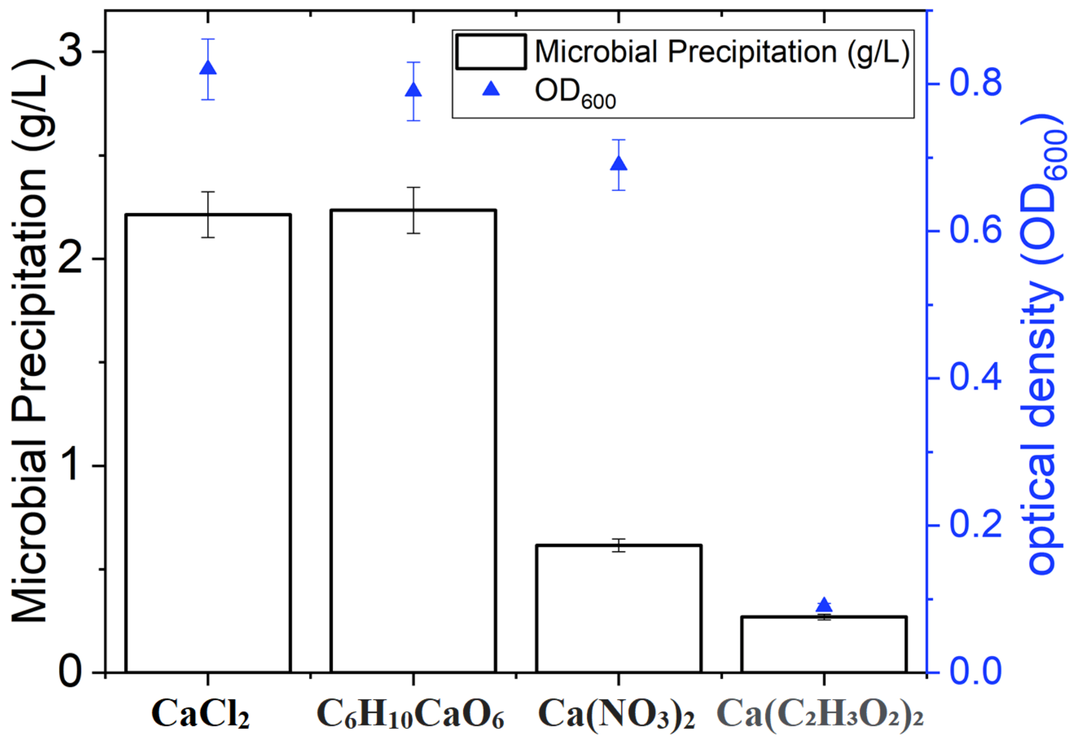

S3 Fig. Evaluation of different calcium sources for microbial induced precipitation in liquid media. Graph shows amount of precipitation after 7 days and optical density after 48h of inoculation with different calcium source; Calcium Chloride ($CaCl_2$), Calcium Lactate ($C_6H_{10}CaO_6$), Calcium Nitrate ($Ca(NO_3)_2$) and Calcium Acetate ($Ca(C_2H_3O_2)_2$).

Optical microscopic experiment:

Evaluation of morphological changes of cell and onset of microbial induced precipitation was performed under Labtech chamber (ibidi, Germany). One ml of SMUC media was placed into the chamber and 10 µl of 24 h grown culture (with OD 0.5) was used as an inoculum, kept for incubation at 30ºC. Images were captured using Leica inverted microscope, initially after every 30 mins of incubation and later every 12 h.

Outcome: One set of experiment was performed to explore the microbial behavior of the induction of precipitation under Labtech chamber in SMUC medium. It was observed that after 24 h of incubation, cells started entangling with each other indicating the commencement of precipitation. After 96 h of incubation, the entire area was covered with induced precipitation. Same result was confirmed with scanning electron microscopic.

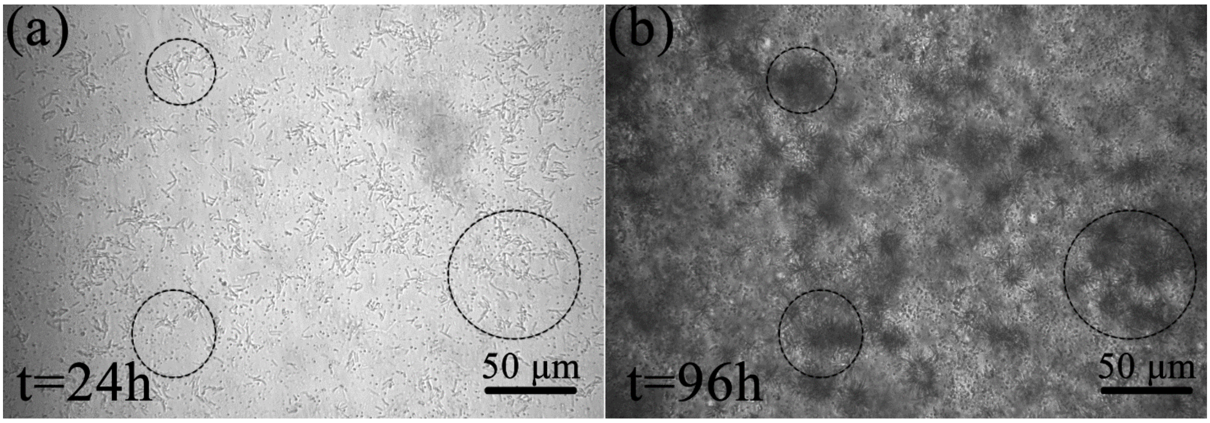

S4 Fig. Optical images of onset of microbial induced precipitation under Labtech chamber. Marked portion in image shows(a) beginning of precipitation and (b) multiple clusters of bacterial induced precipitation.

S1 Tables. Biochemical assay for isolated strains

Bio-chemical Assay	SI1	SI2	SI3	SI4
Melonate	+	+	+	+
Voges Proskauer's	-	-	-	-
Citrate	+	+	+	+
ONPG	-	-	-	-
Nitrate Reduction	+	+	+	+
Catalase	**D**	_	+	+
Arginine	+	+	+	+
Sucrose	-	-	-	-
Mannitol	-	-	-	-
Glucose	-	-	+	-
Arabinose	-	-	-	-
Trehalose	-	-	+	+

Where + represents positive, - represents negative activity and D refers to delayed activity relative to other tested strains, SI – Soil isolates

S2 Table. Blast results: sequences producing significant alignments

Description	Max score	Total score	Query cover	E value	Ident	Accession
Bacillus sp. (in: Bacteria) strain 201705CJKOP-51 16S ribosomal RNA gene, partial sequence	2196	2196	100%	0.0	100%	MG309364.1
Bacillus amyloliquefaciens strain 71 16S ribosomal RNA gene, partial sequence	2196	2196	100%	0.0	100%	MH910175.1
Bacillus velezensis strain Hx05 chromosome, complete genome	2196	19760	100%	0.0	100%	CP029473.2
Bacillus amyloliquefaciens strain PgBE1 16S ribosomal RNA gene, partial sequence	2196	2196	100%	0.0	100%	MH144224.1
Bacillus amyloliquefaciens strain BA31 16S ribosomal RNA gene, complete sequence	2196	2196	100%	0.0	100%	MG548650.1
Bacillus siamensis strain K25.2 16S ribosomal RNA gene, partial sequence	2196	2196	100%	0.0	100%	MH889132.1
Bacillus siamensis strain K23.3 16S ribosomal RNA gene, partial sequence	2196	2196	100%	0.0	100%	MH889128.1
Bacillus siamensis strain K19.1 16S ribosomal RNA gene, partial sequence	2196	2196	100%	0.0	100%	MH889123.1
Bacillus velezensis strain ZF2 chromosome, complete genome	2196	19693	100%	0.0	100%	CP032154.1
Bacillus velezensis strain BIM B-439D chromosome, complete genome	2196	19735	100%	0.0	100%	CP032144.1

S3 Table. Detail data of 2θ (Degree), d spacing (Å), identified phase, hkl and corresponding ICSD references no. for the samples obtained from SMUCG and SMUC treatments.

SMUCG treatment					SMUC treatment				
**2θ (Degree)**	**d spacing (Å)**	**Identified phase**	**hkl**	**ICSD references**	**2θ**	**d spacing (Å)**	**Identified phase**	**hkl**	**ICSD references**
16.02	5.44	Vaterite	012	98-010-9797	16.1	5.44	Vaterite	012	98-010-9797
24.339	3.64	Vaterite	110	98-011-5332	24.339	3.644	Vaterite	110	98-011-5332
26.002	3.42	Vaterite	111	98-010-9797	26.1	3.42	Vaterite	111	98-010-9797
28.146	3.16	Aragonite	110	98-011-0545					
29.328	3.04	Calcite	104	98-002-3929	29.41	3.042	Calcite	104	98-002-3929
31.703	2.82	Calcite	2 0-2	98-000-0099					
32.178	2.77	Aragonite	200	98-011-4649	32.201	2.77	Aragonite	200	98-011-4649
32.416	2.75	Vaterite	116	98-011-5332	33.57	2.67	Vaterite	201	98-010-9797
41.206	2.18	Calcium carbonate	31-2	98-004-0793					
45.482	1.99	Vaterite	0112	98-011-532	46.98	1.94	Vaterite	231	98-010-9797
46.669	1.94	Calcite	018	98-002-1913	47.65	1.90	Vaterite	231	98-010-9796
46.907	1.93	Calcite	024	98-002-1913	49.88	1.82	Vaterite	118	96-000-6092
49.520	1.83	Aragonite	102	98-011-4648	53.69	1.71	Vaterite	222	98-010-9797
64.249	1.44	Calcite	030	98-000-5339					
83.95	1.15	Calcite	134	98-000-5339					
