# Supplementary material for: Microbially induced calcite precipitation using Bacillus velezensis with guar gum
Source: PLoS One. 2020 Aug 12;15(8):e0236745. doi: 10.1371/journal.pone.0236745 (PMC7423064; doi:10.1371/journal.pone.0236745)
Supplement: Attachment — Submitted filename: Response to Reviewers.docx [file pone.0236745.s002.docx]

**Response to the reviewer’s comments**

Manuscript ID: PONE-D-20-07309

**TITLE:** Microbially induced calcite precipitation using *Bacillus velezensis* with guar gum”

**Reviewer #1:**

1. There are different criteria to evaluate the effects of guar gum supplement for calcite precipitation enhanced by *B. velezensis.* The authors describe the idea for the experiment design in the introduction section. The concept for the selection of *B. velezensis* based on the positive traits of urease activity and calcium carbonate precipitation, which conducted good result on guar gum supplement is also briefly described in the introduction section. However, the idea are not stated clearly in the abstract.

Response:

We thank the reviewer for appreciating our work. As per the suggestion, abstract has been revised to provide better clarity on idea which instigated us for carrying out the present work.

Page # 1: We added the following under the ‘Abstract section’ in the revised manuscript.

Mineral precipitation via microbial activity is a well-known process with applications in various fields. This relevance of microbially induced calcite precipitation (MICP) has pushed researchers to explore various naturally occurring MICP capable bacterial strains. The present study was performed to explore the efficiency of microbially induced calcite precipitation (MICP) via locally isolated bacterial strains and role of guar gum, which is a naturally occurring polymer, on the MICP process. The strains were isolated from local soil and screened for urease activity Further, the urease positive strain was subjected to urea and calcium chloride based medium to investigate the efficacy of isolated strain for microbial induced precipitation. as well as microbial precipitation. Among all screened isolates, the soil bacterium that showed urease positive and precipitated calcium carbonate was subjected for 16S rRNA gene sequencing. This strain was identified as *Bacillus velezensis*. Guar gum - a natural polymer, was used as a sole carbon source to enhance the MICP process. Precipitate formed. It was observed that the isolated strain was able to breakdown the guar gum into simple sugars resulting in two-fold increase in calcium carbonate precipitate. Major bio-chemical activities of isolated strain pertaining to MICP such as ammonium ion concentration, pH profiling, and total reducing sugar with time were explored under four different concentrations of guar gum (0.25%, 0.5%, 0.75% and 1% w/v). Maximum ammonium ion concentration (17.5 µg/ml) and increased pH was observed with 1 % guar gum supplementation, which confirms the biological activity. Microstructural analysis of microbial precipitation was performed using scanning electron microscopy (SEM) and X-ray diffraction (XRD) techniques, which confirmed the presence of calcium carbonate in different phases. Further, XRD and SEM based studies corroborated that guar gum supplemented media showed significant increase in stable calcite phase as compared to media without guar gum supplementation results of guar gum supplemented media showed significant increase in the number of calcite peaks as compared to media without guar gum supplementation. Significant diverse group of nitrogenous compounds were observed in guar gum supplemented medium when subjected to Gas Chromatography–Mass spectrometry (GC-MS) profiling.

2. Primers for 16S rRNA gene sequencing should have a reference cited. How about the similarity to the known *Bacillus velezensis*?

Response:

Reference used for the 16S rRNA gene sequencing has been added in the revised manuscript.

To explore similarity/difference with Bacillus velezensis, whole genome sequencing is planned as a separate study. In present work we have not performed any comparative study with the known Bacillus velezensis strain. However, this strain is reported in literature as plant growth promoting rhizobacterium and has not been explored as an ureolytic strain and its potential for MICP, which is the main objective of this work.

The following new references are added to the revised manuscript:

- Saiki RK, Gelfand DH, Stoffel S, Scharf SJ, Higuchi R, Horn GT, et al. Primer-directed enzymatic amplification of DNA with a thermostable DNA polymerase. Science (80- ). 1988;239: 487–491.
- Walker K. Identification of Bacterial Species Using Colony PCR. 2015.

3. Figure titles are not necessary included in the results.

Response:

As per the journal guidelines, the figure titles are placed after each para where they are referred. In the figure legends, bacterial strain should be indicated. Is the same for the experiments done? The consistence of materials used is very important.

Response:

We have revised the figure legends to maintain consistency. Yes, throughout the work the same bacterial strain i.e. *Bacillus velezensis* was used.

**Response to the reviewer’s comments**

Manuscript ID: PONE-D-20-07309

**TITLE:** Microbially induced calcite precipitation using *Bacillus velezensis* with guar gum”

**Reviewer #2:**

1. Please clarify what “the green brick” refers to. Should it not be “a green brick”? Also, in the same sentence, it is likely the urea present in urine that is of interest and not the other nutrients. Please change and edit. Also, clarify what “it” refers to in the next sentence.

Response:

The said correction has been made in the revised manuscript. The missing keyword ‘urea’ has been incorporated in the subsequent sentence. The word ‘it’ refers to ‘urine’ and corresponding corrections are also made in the revised manuscript.

Page # 2: We added the following under the ‘Introduction section’ in the revised manuscript.

Urine is a rich source of urea and contains nutrients like nitrogen, potassium and phosphorus too [25]. Therefore, recyclable urine can be a potential alternative source of urea for MICP mediated applications. Strains belonging to Bacillus genus are suitable for MICP applications as they can tolerate adverse environmental stresses such as alkaline pH [26].

2. You mention “strains belonging to *Bacillus*…such as alkaline pH” but there is no reference for this. You therefore might want to add this reference: <https://www.sciencedirect.com/science/article/abs/pii/S2213343718304251>

Response:

Above suggested reference has been incorporated in the revised manuscript.

3. Change “Since medium pH” to “Since the medium pH”

Response:

We thank the reviewer for suggestion, this grammatical error has been rectified in the revised manuscript.

4. Screening for urea hydrolysis: it is not clear from this section how the concentration of urea and ammonium ions were measured once urea had hydrolysed. Please clarify.

Response:

Since, the objective of the planned experiment was to screen urease positive strain from the soil isolated bacterial strains, the strains were subjected to urea-based agar medium ( medium composition; 0.1g glucose, 0.1 g peptone, 0.5 g NaCl, 0.2 g mono-potassium phosphate, 2 g urea, 0.0012 g phenol red dye and 2 g agar in 100 ml distilled water) for qualitative analysis. For strains which can hydrolyse urea, the colour of the medium will turn into dark pink after 24 hrs of incubation. The medium pH shifts towards alkaline after urea hydrolysis as a result of release of ammonium ion. Phenol red dye, supplemented in the medium, acts as pH indicator. Organism unable to hydrolyse urea will not affect the medium colour. In the quoted section, the identification of urease positive strain is done only based on the change in medium colour.

5. **“**Further, qualitative analysis based on pH indicator (phenol red) for urea degradation” – was the urea degradation not quantified by measuring the concentration of ammonium ions, and if not, why not?

Response:

pH indicator (phenol red) based approach is ideal for qualitative analysis for rapid screening of positive strains. Urea based medium was used in the present study, where only urea hydrolysis is expected to result in a change of medium pH. Hence, for screening qualitative analysis was performed. This can be seen from **figure S1 reproduced below** (previously figure 1 of main manuscript), where it shows that only one isolated strain (*B. velezensis)* was grown and a change in medium colour was observed. Hence at this point, no quantitative estimation was deemed necessary.

As far as quantitative estimation for urea hydrolysis is concerned, we have used Nessler’s reagent assay for post-screening investigations. After screening, once we found the urease positive bacterial strain, we performed qualitative estimation of urea hydrolysis to optimize the urease activity in terms of optimum temperature and pH before the start of MICP experiments

Figure S1; Agar column showing urea hydrolysis after 24h of Soil Isolated strains (a) SI1, (b) SI2, (c) SI3, (d) SI4, (e) C (without organism). SI1 shows colour change due to increase in medium pH by bacterial activity.SI1; soil isolate 1, SI2; soil isolate 2, SI3; soil isolate 3, SI4; soil isolate 4.

6. Please confirm what the calcium concentration was and if this was the same for all bars shown in the graph.

Response:

The calcium concentration used was 25 mM and it was same for all the cases.

7. **“**Aragonite was not found in precipitation in SMUC medium” – please mention why this might be the case.

Response:

On careful examination of XRD pattern, we found that there is one peak that corresponds to Aragonite in the SMUC medium (at 2θ value of 32.178 with hkl value 200) matching with ICSD reference 98-011-4649. This peak with same diffraction angle and d-spacing (2.77) was observed in SMUCG treatment too. However, there were additional peaks for Aragonite observed in SMUCG treatment probably due to the effect of guar gum supplementation in the medium. Similar trend was observed with calcite peaks too.

It is also reported in literature that combinations of inorganic molecule and organic macromolecules provide suitable hybrid materials to control the structures, morphologies and properties of calcium carbonate precipitates. [https://doi.org/10.1002/1521-4095(200010)12:20<1543::AID-ADMA1543>3.0.CO;2-P](https://doi.org/10.1002/1521-4095(200010)12:20%3C1543::AID-ADMA1543%3E3.0.CO;2-P); <https://doi.org/10.1039/B003334K>; DOI: 10.1021/acssuschemeng.8b01658

A detailed XRD pattern for both the treatments is provided in Table S3 additionally incorporated as part of supplementary material. The results of XRD for both the treatments are also updated and incorporated in the revised manuscript. Fig. 5 has also been updated in the revised manuscript for better comparative view between the treatments.

8. Figure 6a: should it not be ammonium ions on the y-axis?

Response: The reviewer is correct. It should be ammonium ion concentration on the y axis.

Page # 22: Figure 6 has been rectified and placed in revised manuscript under ‘Figure and caption section’ as following:

Figure 6: Exploration of microbial physiology under flask condition with guar gum additive. (a) temporal evolution of ammonium concentration in SMUCG media and temporal evolution of pH in SMUCG media (inset) (b) change in amount of total reducing sugar in SMUCG media with time compared with SMUC medium (0% curve). Legend shows guar gum percentage (w/v) in SMUCG media (Error bars represents the standard deviation of the data of three independent experiments).

9. “…known to be the most stable form of calcium carbonate [39]" – it might be stable but it might also not be the strongest form. Please add some literature here about the strength of the different calcium carbonate types.

Response:

Major forms of calcium carbonate precipitate are calcite, aragonite and vaterite of which vaterite and calcite are the most common bacterial calcium carbonate polymorphs. Calcite is known to be the most stable form of calcium carbonate. However, it is reported that aragonite and vaterite respectively have greater hardness than calcite. The reported hardness is 3.4 to 0.1 GPa for calcite, 4.2 to 0.3 GPa for vaterite and 4.2 to 0.01 GPa, for aragonite respectively [Ref. <https://doi.org/10.1007/s10853-017-1884-x>].

10. guar gum as an economical substrate for bacterial cultivation” – mention is made of ‘economics’ but no economic value or cost of guar gum is given. Please add this or revise this sentence.

Response:

India tops the cultivation of guar, producing more than 850,000 tons amounting to 80% of the total guar produced all over the world. The price of guar gum in India ranges from 1-1.5 US dollar per kilogram.

Page # 3: Content has been incorporated in the revised manuscript under the Introduction section as following:

In this study, Guar gum (*Cyamopsistetragonolobus*, family Leguminosae) which is a green, non-toxic, easily available, cost-effective and easily biodegradable polysaccharide-based natural polymer[33][28] was explored for its role on MICP process. India tops the cultivation of guar producing more than 850,000 tons and amounting to 80% of the total guar produced all over the world [34].

The following new references are added to the revised manuscript:

- Henze J, Randall DG. Microbial induced calcium carbonate precipitation at elevated pH values (> 11) using *Sporosarcina pasteurii.* J Environ Chem Eng. 2018;6: 5008–5013.
- Ševčík R, Šašek P, Viani A. Physical and nanomechanical properties of the synthetic anhydrous crystalline CaCO 3 polymorphs: vaterite, aragonite and calcite. J Mater Sci. 2018;53: 4022–4033.
- Antil M, Chhikara S. Guar Korma: A Good Alternate to Replace Groundnut Cake in the Diet of Buffalo Calves: A Review. Int J Curr Microbiol App Sci. 2019;8: 1805–1816.
- Kato, T. Polymer/calcium carbonate layered thin-film composites. Adv. Mater. 2000, 12, 1543−1546.
- Falini, G.; Fermani, S.; Gazzano, M.; Ripamonti, A. Polymorphism and architectural crystal assembly of calcium carbonate in biologically inspired polymeric matrices. J. Chem. Soc., Dalton Trans. 2000, No. 21, 3983−3987.

**Response to the reviewer’s comments**

Manuscript ID: PONE-D-20-07309

**TITLE:** Microbially induced calcite precipitation using *Bacillus velezensis* with guar gum”

**Reviewer #3:**

1.Vaterite and calcite crystals are spherical and rhombohedral structure, respectively. Why are these structures not seen in the SEM micrographs? Sometimes, bacterium holes can be seen in the crystal surface, why not here?

Response:

We have now supplied additional SEM micrographs where vaterite and calcite crystals are present and bacterium holes as well. Further, we have added the pertinent texts in the revised manuscript.

Figure 3 of the manuscript which demonstrated SEM images and EDS data for both the treatments (SMUC and SMUCG) is now split to address both the treatments separately. Figure 3 (a-b) now depicts SMUC treatment while Figure 4 (a-d) illustrates SMUCG treatment.

SEM micrographs of the samples from SMUC medium is shown in Fig. 3a. Several randomly embedded spherical meta-stable vaterite crystals [Ref. 1: https://doi.org/10.1007/s10853-017-1884-x // Ref. 2: RSC Advances, 2012, 2, 10015–10019, DOI: 10.1039/c2ra20975f] on the bed of nano-crystalline flower-petal like aragonites [Ref. 1: https://doi.org/10.1007/s10853-017-1884-x // Ref. 2: MATEC Web of Conferences 159, 01054 (2018) https://doi.org/10.1051/matecconf/201815901054] are observed. Further, Figs. 4 (a-c) depict the SEM micrographs of the samples from SMUCG medium. Figure 4b depicts the stacking of rhombohedral and stable calcite crystals as marked. Further, sites with holes due to bacterium also often found are shown in Fig. 4c as reported by others as well https://doi.org/10.1371/journal.pone.0210339. The existence of stable calcite crystals found higher after adding gaur gum from the SEM based studies is also further confirmed by XRD studies elaborated later.

\# Page 8: The following revision is made and added in revised manuscript:

Figures 3 (a-b) show the SEM images and EDS data of calcium carbonate precipitates obtained with SMUC treatment while Figure 4 (a-d) depict the SEM images with SMUCG treatment. With SMUC, in absence of guar gum supplementation, microbial induced nanocrystalline precipitates were observed (Fig. 3(a)). Several randomly embedded spherical meta-stable vaterite [39,40] crystals are seen on the bed of nanocrystalline flower-petal like aragonite [39,41] (Fig. 3(b)). On the other hand, multi-shaped densely populated sharp pointed flaked precipitates were observed in case of SMUCG treatment supplemented with guar gum (Fig 4a). Figure 4(b) depicts the stacking of stable calcite [40,42] crystals. The presence of bacteria and its biological activity mediated MICP process was further established in Fig. 4(c) where precipitates sites with numerous holes are found. These observed impressions on the surface of precipitate indicate the presence of bacterially mediated MICP process [6]. The existence of stable calcite crystals found higher after adding gaur gum from the SEM based studies is also further confirmed by XRD studies appended later. The precipitates were then subjected to spectral mapping of elements such as carbon, oxygen and calcium to characterize the calcium carbonate precipitation by bacterial strains. The EDS data of SMUC and SMUCG treatments shown in Fig. 3(b) and Fig. 4(d), respectively indicate the presence of calcium, carbon and oxygen which are the constituents of CaCO_3_. This was further confirmed by XRD analysis.

Figure 3 SEM images of *Bacillus velezensis* mediated precipitated crystals with SMUC medium (a) showing randomly embedded spherical meta-stable calcium carbonate crystals on the bed of flower-petal like nanocrystalline phase (inset: higher magnification image) and (b) elemental distribution of Carbon (C), Oxygen (O) and Calcium (Ca). (Incubation period was 7 days in all cases.)

Figure 4 SEM images of *Bacillus velezensis* mediated precipitated crystals with SMUCG medium (a) showing calcium carbonate precipitates with different shapes, (b) showing stacking of rhombohedral and stable calcite crystals and (c) showing perceptible holes along with bacteria which indicates bacterial induced precipitation. (d) Elemental distribution of Carbon (C), Oxygen (O) and Calcium (Ca). (Incubation period was 7 days in all cases.)

2. Can you obtain percentages of vaterite and calcite using Rietveld refinement? It is important to know crystal proportion to evidence effect of guar gum in the process.

Response:

We appreciate the proposal given to carryout Rietveld refinement analysis. However, errorless Rietveld analysis is only possible where sharp, distinct and highly crystalline XRD peaks are observed. For example, Rietveld analysis is reported for highly crystalline calcium carbonate polymorphs synthesized by conventional chemical route. <https://doi.org/10.1007/s10853-017-1884-x>; <https://doi.org/10.1111/jace.14023> ; <https://doi.org/10.1051/matecconf/201815901054> doi.org/10.26434/chemrxiv.11364857.v1

However, in the present XRD data, we observe the presence of amorphous content, peak broadening (possibly due to nano-crystalline phases) and overlapped peaks (two or more polymorph closely co-exists and possibly due to residual stress) which are common features for the formation of calcium carbonate through microbial route. Thus, in the present, investigation regarding Rietveld refinement is not attempted. However, in future this study would be taken up as a separate individual work as it requires special attention. To get high crystallinity in the microbial induced calcium carbonate precipitates, further post processing such as controlled annealing might be required. It is expected at desired temperature bio components will be removed from the samples and crystallinity would be improved due to further crystal growth which is in-general established for the ceramic materials. Thus, Rietveld analysis requires extensive work, which is outside the scope of the current manuscript and will be attempted in a future work. We appreciate the reviewer’s insight on this issue.

A comprehensive table (Table S3) is now added in revised manuscript where experimental ‘d spacing’ and ‘2θ’ are compared with corresponding ICSD references. This thorough data analysis will qualitatively establish the increase in stable calcite phase since we added gaur gum. Further, in XRD plot absolute intensity values are now appended to understand clearly the increase in peak intensity after the addition of gaur gum.

#Page 9: The revision made and added in revised manuscript as following:

Thoroughly indexed XRD patterns of dried and crushed precipitated crystals obtained from both the treatments (SMUC and SMUCG) are shown in Fig. 5 and Fig. 5 (inset)). Detail data of corresponding 2θ, d spacing, hkl, phase and the ICSD reference no. are summarized in Table S3 as supplementary material. Figure 5 clearly suggests that different polymorphs of calcium carbonate co-exist in both the samples. However, the presence of calcite peaks (Fig. 5, inset and Table S3) and their relative intensities (Fig. 5) in SMUCG treatment are significantly higher as compared to SMUC treatment. Therefore, it would qualitatively establish the fact that there is a significant increase in stable calcite phase after gaur gum supplementation in the growth medium.

Figure 5 A comparative view of XRD pattern of *Bacillus velezensis* induced precipitates under guar gum (SMUCG) and without guar gum (SMUC) supplemented media compositions. Inset figure is showing indexed XRD data with corresponding planes for both the treatments. XRD pattern of *Bacillus velezensis* induced precipitates under guar gum (SMUCG) and without guar gum (SMUC) supplemented media compositions. In the spectrum, V represents vaterite, A for aragonite, and C for calcite and CC for calcium carbonate. Incubation period was 7 days in all cases.

Tables S3: Detail data of 2θ, d spacing, identified phase, hkl and corresponding ICSD references no. for the samples obtained from SMUCG and SMUC treatments.

| **SMUCG treatment** | | | | | **SMUC treatment** | | | | |
| --- | --- | --- | --- | --- | --- | --- | --- | --- | --- |
| **2θ** | **d spacing** | **Identified**  **Phase** | **hkl** | **ICSD references** | **2θ** | **d spacing** | **Identified**  **phase** | **hkl** | **ICSD references** |
| 16.02 | 5.44 | Vaterite | 012 | 98-010-9797 | 16.1 | 5.44 | Vaterite | 012 | 98-010-9797 |
| 24.339 | 3.64 | Vaterite | 110 | 98-011-5332 | 24.339 | 3.644 | Vaterite | 110 | 98-011-5332 |
| 26.002 | 3.42 | Vaterite | 111 | 98-010-9797 | 26.1 | 3.42 | Vaterite | 111 | 98-010-9797 |
| 28.146 | 3.16 | Aragonite | 110 | 98-011-0545 |  |  |  |  |  |
| 29.328 | 3.04 | Calcite | 104 | 98-002-3929 | 29.41 | 3.042 | Calcite | 104 | 98-002-3929 |
| 31.703 | 2.82 | Calcite | 2 0-2 | 98-000-0099 |  |  |  |  |  |
| 32.178 | 2.77 | Aragonite | 200 | 98-011-4649 | 32.201 | 2.77 | Aragonite | 200 | 98-011-4649 |
| 32.416 | 2.75 | Vaterite | 116 | 98-011-5332 | 33.57 | 2.67 | Vaterite | 201 | 98-010-9797 |
| 41.206 | 2.18 | Calcium carbonate | 31-2 | 98-004-0793 |  |  |  |  |  |
| 45.482 | 1.99 | Vaterite | 0112 | 98-011-532 | 46.98 | 1.94 | Vaterite | 231 | 98-010-9797 |
| 46.669 | 1.94 | Calcite | 018 | 98-002-1913 | 47.65 | 1.90 | Vaterite | 231 | 98-010-9796 |
| 46.907 | 1.93 | Calcite | 024 | 98-002-1913 | 49.88 | 1.82 | Vaterite | 118 | 96-000-6092 |
| 49.520 | 1.83 | Aragonite | 102 | 98-011-4648 | 53.69 | 1.71 | Vaterite | 222 | 98-010-9797 |
| 64.249 | 1.44 | Calcite | 030 | 98-000-5339 |  |  |  |  |  |
| 83.95 | 1.15 | Calcite | 134 | 98-000-5339 |  |  |  |  |  |

3. How was microbiological precipitation measured in the Figure S1?

Response:

After incubation period, samples were centrifuged and supernatant was discarded. This was followed by drying at 45 °C for 24 hrs. Dried precipitates were quantified on weighing machine.

4. Where is the microorganism shown to grow from 15 to 45 °C?

Response:

Data was recorded for bacterial growth on varied range of temperatures but was not presented in the earlier manuscript. For sake of clarity and completeness, the text has been incorporated in revised manuscript under subsection ‘Screening and identification of strains’ and figure S2 in supplementary material.

Page # 7: The following text has been added in the revised manuscript.

To characterize the bacterial growth on varied range of temperature, strain was inoculated in SMU media and kept for incubation at different temperatures (15, 20, 25, 30, 35, 40 and 45 °C) and OD was measured after 24 hrs of each incubation. It was observed that *Bacillus velezensis* was able to grow on the experimented temperature range and optimum growth was observed at 30 °C. There was no significant difference observed in OD between 30 and 35 °C. Minimum growth was observed at 15 °C as presented in Fig S2.

Figure S2 Temporal evolution of bacterial growth under varied range of temperature (15-45 °C)

**5.** In the last paragraph of page 10, the conclusion “This may be the probable reason for higher ammonium ion concentration observed with medium supplemented with guar gum” should be revised. The higher ammonium ion concentration is due urea hydrolysis with or without guar gum, for that reason, the presence or formation of different detected metabolites should be explained better.

Response:

The said paragraph is modified to provide better explanation in the revised manuscript.

Page # 11: Modified text has been added in the subsection of ‘Bacterial physiology under guar gum supplementation’ in the revised manuscript as following:

In SMUCG treatment, more fused ring aromatic and nitrogenous compounds such as Methanone, (1-hydroxycyclohexyl)phenyl-, 4H-Benzopyran-3-carbonitrile, 2-amino-5-hydroxy-4-phenyl-, Uracil, 5-acetyl-6-amino-1,3-dimethyl, Hex-5-enylamine, L-5-Hydroxytryptophan, tert-butyldimethylsilyl ether, tert-butyldimethylsilyl ester, Benzene, 1-nitro-3-(2-phenylethenyl were observed as compared to SMUC treatment.

These additional organic macromolecules found in guar gum supplemented medium could be the probable reason for the enhancement of microbial metabolic activity leading to augmentation of the MICP process. Organic macromolecules are known to facilitate the biomineralization phenomenon by regulating its dynamic process (nucleation site, crystal growth etc) or by providing a structural framework to initiate the process [28-32]. Same observation was made in SMUCG treatment as well.

6. I recommended placing Figure 1 to supplementary material because it is not enough important to put in the principal page of the paper.

Response:

As per suggestion, figure has been placed in supplementary material**.**

The following new references are added to the revised manuscript:

- Chaparro-Acuña SP, Becerra-Jiménez ML, Martínez-Zambrano JJ, Rojas-Sarmiento HA. Soil bacteria that precipitate calcium carbonate: mechanism and applications of the process. Acta Agronómica. 2018;67: 277–288.
- Ševčík R, Šašek P, Viani A. Physical and nanomechanical properties of the synthetic anhydrous crystalline CaCO 3 polymorphs: vaterite, aragonite and calcite. J Mater Sci. 2018;53: 4022–4033.
- Saikia J, Saha B, Das G. Morphosynthesis of framboidal stable vaterite using a salicylic acid-aniline dye as an additive. RSC Adv. 2012;2: 10015–10019.
- Putranto WA, Umardhani Y, Sulistyo, Yurianto, Bayuseno AP. Analysis of calcium carbonate polymorphs deposited in water piping system and the effect of tartaric acid additive. MATEC Web Conf. 2018;159. Available: https://doi.org/10.1051/matecconf/201815901054
- Kim Y-Y, Schenk AS, Ihli J, Kulak AN, Hetherington NBJ, Tang CC, et al. A critical analysis of calcium carbonate mesocrystals. Nat Commun. 2014;5: 1–14.

**Response to Academic Editor’s comments**

**Manuscript ID: PONE-D-20-07309**

**TITLE:** Microbially induced calcite precipitation using *Bacillus velezensis* with guar gum”

1. Please ensure that your manuscript meets PLOS ONE's style requirements, including those for file naming. The PLOS ONE style templates can be found at

<https://journals.plos.org/plosone/s/file?id=wjVg/PLOSOne_formatting_sample_main_body.pdf> and

<https://journals.plos.org/plosone/s/file?id=ba62/PLOSOne_formatting_sample_title_authors_affiliations.pdf>

Response:

It has been taken care while preparing the revised manuscript

2. Please ensure that you refer to Figure 3 in your text as, if accepted, production will need this reference to link the reader to the figure.

Response:

All figures have been cited in the revised manuscript accordingly.

3. Please include captions for your Supporting Information files at the end of your manuscript, and update any in-text citations to match accordingly. Please see our Supporting Information guidelines for more information: <http://journals.plos.org/plosone/s/supporting-information>

Response:

Caption file for supporting material has been added in the revised manuscript
